# Characteristics of men responding to an invitation to undergo testing for prostate cancer as part of a randomised trial

**DOI:** 10.1186/s13063-016-1624-6

**Published:** 2016-10-13

**Authors:** Eleanor I. Walsh, Emma L. Turner, J. Athene Lane, Jenny L. Donovan, David E. Neal, Freddie C. Hamdy, Richard M. Martin, and the CAP & ProtecT Trial Groups, Investigators, Richard Martin, Jenny Donovan, David Neal, Freddie Hamdy, Emma Turner, Chris Metcalfe, Jonathan Sterne, Sian Noble, Research staff, Liz Hill, Siaw Yein Ng, Naomi Williams, Liz Down, Eleanor Walsh, Joanna Thorn, Charlotte Davies, Laura Hughes, Mari-Anne Rowlands, Lindsey Bell, Management Committee, Emma Turner, Richard Martin, Jenny Donovan, Chris Metcalfe, Jonathan Sterne, Sian Noble, Yoav Ben-Shlomo, Athene Lane, Steven Oliver, Peter Brindle, Simon Evans, CAP & ProtecT Trial Steering Committee, Michael Baum, Peter Albertsen, Tracy Roberts, Mary Robinson, Jan Adolfsson, David Dearnaley, Anthony Zeitman, Fritz Schröder, Tim Peters, Peter Holding, Teresa Lennon, Sue Bonnington, Malcolm Mason, Jon Oxley, Richard Martin, Jenny Donovan, David Neal, Freddie Hamdy, Emma Turner, Athene Lane, Data Monitoring Committee, Lars Holmberg, Robert Pickard, Simon Thompson, Usha Menon, Peter Albertsen, Colette Reid, Jon McFarlane, Jon Oxley, Mary Robinson, Jan Adolfsson, Michael Baum, Anthony Zeitman, Amit Bahl, Anthony Koupparis, Administrative staff, Marta Tazewell, Genevieve Hatton-Brown

**Affiliations:** 1 School of Social and Community Medicine, University of Bristol, Canynge Hall, 39 Whatley Road, Bristol, BS8 2PS UK; 2 Nuffield Department of Surgical Sciences, John Radcliffe Hospital, Oxford, OX3 9DU UK

**Keywords:** Prostate cancer, Screening, Randomised controlled trial, PSA testing, Prostate specific antigen testing

## Abstract

**Background:**

Sociodemographic characteristics are associated with participating in cancer screening and trials. We compared the characteristics of those responding with those not responding to a single invitation for prostate-specific antigen (PSA) testing for prostate cancer as part of the Cluster randomised triAl of PSA testing for Prostate cancer (CAP).

**Methods:**

Age, rurality and deprivation among 197,763 men from 271 cluster-randomised primary care centres in the UK were compared between those responding (*n* = 90,300) and those not responding (*n* = 100,953) to a prostate cancer testing invitation.

**Results:**

There was little difference in age between responders and nonresponders. Responders were slightly more likely to come from urban rather than rural areas and were slightly less deprived than those who did not respond.

**Conclusion:**

These data indicate similarities in age and only minor differences in deprivation and urban location between responders and nonresponders. These differences were smaller, but in the same direction as those observed in other screening trials.

**Trial registration:**

ISRCTN92187251. Registered on 29 November 2004.

## Background

Screening for prostate cancer (PCa) has been shown to reduce disease-specific morbidity and mortality through early detection, but at the expense of overdiagnosis and overtreatment of indolent cancer [1–3]. The UK National Screening Committee (UKNSC) does not currently recommend population screening for PCa, although testing can be performed on request [4]. The UKNSC awaits the results of ongoing nationwide trials to further inform UK PCa screening policy. The Cluster randomised triAl of PSA testing for Prostate cancer (CAP) is an effectiveness trial comparing PCa-specific mortality in men invited (intervention arm) and men not invited (control arm) to prostate-specific antigen (PSA) testing in primary care [5] and the Prostate testing for cancer and treatment (ProtecT) randomised controlled trial (RCT), which evaluates the effectiveness and cost-effectiveness of treatment for localised, PSA-detected PCa [6].

Material and social deprivation is associated with lower rates of attending cancer screening programmes for both men [7–9] and women [10–14], as well as RCT participation [15]. Failure to enroll participants who are representative of the target population can compromise the generalisability of trial findings [16], for example, due to a healthy volunteer effect (HVE) [17] with younger, healthier, more-educated individuals taking part compared with nonattendees [18]. Here we compare the characteristics of those who responded to the invitation to take part in the intervention arm of the CAP trial with those who did not, using routine data available from primary care centres.

## Methods

**Fig. 1:**
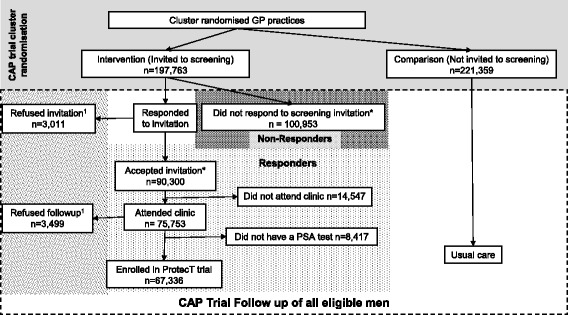
Trial and follow-up organisation

Men aged 50–69 years from 271 primary care (GP) practices in England and Wales were cluster-randomised to receive the intervention in the CAP trial (Fig. 1) of a postal invitation to attend a one-off appointment for a PSA test screening for PCa. Those who returned a reply slip by post accepting this invitation (i.e. ‘responders’) were given an appointment and invited to participate in the screening and diagnostic stage of the ProtecT trial [6]. Responders who were ineligible or decided not to participate in the ProtecT study were followed up as part of the CAP trial (discussed in full elsewhere; [5]). Those who declined the initial invitation (i.e ‘nonresponders’) were also followed up as part of the CAP trial [5].

### Data

Date of birth and postcode were provided by the primary care practices for all men who were invited to screening for recruitment purposes. These are the only demographic data items available for both responders and nonresponders due to governance and ethical permissions. Postcode was used to calculate the Index Multiple Deprivation (IMD) score and the Rural and Urban Area Classification (RUAC).

### Index Multiple Deprivation (IMD)

The IMD is a widely used measure of area deprivation in England and Wales and has been shown to represent deprivation appropriately for both urban and rural areas [19]. The IMD is an overall composite score of weighted domains with higher scores indicating more deprivation encompassing aspects of socioeconomic status including unemployment, claiming financial support from the state, poor health, low educational attainment, criminal victimisation, household condition and overcrowding. English 2004 and Welsh 2005 IMD scores are not directly comparable and are, therefore, analysed separately [20].

### Rural and Urban Area Classification (RUAC)

The RUAC (2004) is a measure of population density and sparseness in the UK. For RUAC, areas of more than 10,000 people are considered to be urban, otherwise they are classified as rural (i.e. less than 10,000 people).

### Statistical analysis

A *t* test was used to compare mean age and deprivation in responders and nonresponders. Odds ratios (ORs) were used to compare the proportion of responders and nonresponders living in an urban location. Gartner et al. [19] found that deprivation accounted for the differences they found in outcomes by urban-rural location, and we therefore adjusted this analysis by IMD score. Analyses were conducted in STATA version 13.

## Results

Of 197,763 men registered at primary care practices who were randomised to receive invitations to the PSA testing clinic, 46 % (*n* = 90,300) accepted the invitation (responders) and 51 % did not respond (*n* = 100,953; i.e. nonresponders). The remaining men either explicitly refused the invitation (1.5 %; *n* = 3,010) or, having attended, refused to take part in the trial (1.8 %; *n* = 3,499). Eighty-four percent (*n* = 75,753) of responders ultimately attended the clinic, of which 98 % (*n* = 65,836) reported themselves as being of White ethnic origin.

**Fig. 2:**
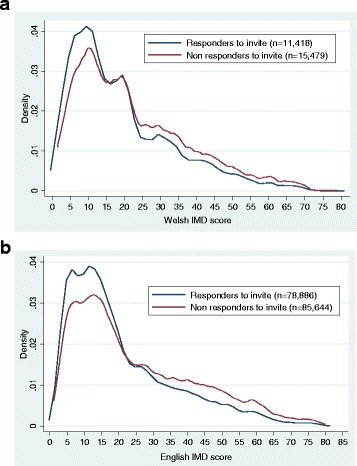
**a** Frequency distribution of Welsh Index Multiple Deprivation (IMD) scores for those responding (solid line) to a one-off invite to a PSA test compared to those who did not respond (dash) to this invitation. **b** Frequency distribution of English IMD scores for those responding (solid line) to a one-off invite to a PSA test compared to those who did not respond (dash) to this invitation

The mean age of both responders and nonresponders was 59 years (5.47 and 5.66 standard deviations (SDs), respectively; see Table 1). Responders in England and Wales were less deprived on average compared to nonresponders (IMD 20.66 (SD 15.49) versus 25.04 (SD 17.74); *p* < 0.001 and 19.04 (SD 14.08) versus 22.27 (SD 15.28); *p* < 0.001, respectively). The distribution of IMD scores between responders and nonresponders are comparable (see Fig. 2a and b), as demonstrated by the interquartile range (IQR) of both IMD scores in England (responders’ IQR 9.3–28.5 versus nonresponders’ IQR 10.9–37.2) and Wales (responders’ IQR 8.2–26.9 versus nonresponders’ IQR 10.3–31.8).

**Table 1:** Demographics of responders and nonresponders to screening invitation

	Nonresponders	Responders	*p* value for difference
	*n* = 100,953	*n* = 90,300	
Age at randomisation (SD)	58.9 (5.66)	59.1 (5.47)	< 0.0001^a^
*N*	100,953	90,285	
Mean England Index of Multiple Deprivation (IMD) score (SD)	25.04 (17.74)	20.66 (15.49)	< 0.0001^a^
*N*	85,521	78,882	
Mean Wales Index of Multiple Deprivation (IMD) score (SD)	22.27 (15.28)	19.04 (14.08)	< 0.0001^a^
*N*	15,432	11,418	
Urban (>10,000 people)			
England and Wales combined % OR (95 % CI)	87 % (baseline)	85 %	< 0.0001^c^
		0.84 (0.82–0.86)	
England % (OR; 95 % CI)	86 % (baseline)	84 % (0.85; 0.83–0.87)	< 0.0001^c^
[adjusted OR; 95 % CI; *p* value]^b^		[1.04; 0.99–1.09]	[0.14]^c^
Wales % (OR; 95 % CI)	95 % (baseline)	95 % (1.01; 0.91–1.13)	0.83
[adjusted OR; 95 % CI; *p* value]^b^		[1.11; 0.95–1.30]	[0.19]^c^

^a^ *t* test, ^b^English and Welsh IMD scores are adjusted for separately because they are derived differently and should not be combined, ^c^Logistic regression

*CI* confidence interval, *OR* odds ratio

Responders were also slightly less likely to live in urban areas with a population of more than 10,000 than nonresponders (85 % [95 % confidence interval (CI) 0.85 to 0.86] versus 87 % [95 % CI 0.87 to 0.87]; *p* < 0.001), and slightly more likely to be from urban locations when controlling for deprivation, in both England (crude OR 0.85, 95 % CI 0.83 to 0.87 compared with adjusted OR 1.04, 95 % CI 0.99 to 1.09) and Wales (crude OR 1.01, 95 % CI 0.91 to 1.13 compared with adjusted OR 1.11, 95 % CI 0.95 to 1.30).

There was no difference in the results when classifying those who accepted the invitation, but did not ultimately attend the clinic or undergo a PSA test (*n* = 22,964), as nonresponders rather than responders.

## Discussion

There were very small differences between those who responded and those who did not respond to a single invitation to receive a PSA test as the intervention in the CAP trial. Responders and nonresponders were similar in age. Responders were only slightly less deprived than the nonresponders, and there were negligible differences in urban-rural location. Although we report small *p* values, these reflect the large numbers in the study as the absolute differences are very small in magnitude.

The CAP trial gained consent after randomisation to receive or not receive an invitation to screening and, by analysing on this intention-to-screen basis, reduces the risk of self-selection bias influencing the representativeness to the target population. This is compared to other trials where consent was gained prior to randomisation [21]. However, differences between men who did and did not actually undergo a PSA test is a key issue for policy-makers and cannot be ruled out as having an impact on the effectiveness of screening.

It was only possible to derive age, urban/rural location and an ecological measure of deprivation from the available data because of ethical and governance regulations. The large randomly allocated cohort and intention-to-screen analysis in the CAP trial helps to mitigate the effect of unmeasured factors on generalisability of the trial. While the responders and nonresponders were similar according to the characteristics measured, it cannot be ruled out that there were other differences between the groups. Important differences in comorbidities, education, income and decision-making between attendees and nonattendees have been shown elsewhere [32, 33] and remain key factors for policy-makers.

We are unable to comment on the impact of any post-randomisation differences between the groups on mortality and PCa incidence. Previous trials have suggested that nonparticipation in the screening arm is associated with higher mortality [22] and that controlling for ‘healthier’ attenders in the screening arm is important to avoid overestimating the effect of screening on mortality [18]. This is something that could be investigated in the future once the median 10-year intention-to-screen analyses have been reported.

This RCT invited over 190,000 patients to attend a one-off blood test from different geographical regions across the UK. According to these characteristics, those who responded to the invitation were representative of the population who were invited as a whole and comparable with those enrolled in other screening trials [23, 24]. Compared to other PCa screening trials, considerably more men were invited in this trial and overall response rates were studied [6]. Other trials have not consistently reported the numbers initially invited (cf. [25–31]) or have reported on a sample of participant uptake of invitations to testing prior to publication of the primary analysis [21].

Group-level data (i.e. postcode) limits the sensitivity of deprivation data at an individual level, and area measures of deprivation are an average of the overall deprivation of the population, which arguably cannot represent the level of deprivation in the age- and gender-specific group included in this trial [34]. Further, without ethnicity information for all those invited we cannot investigate the lack of ethnic diversity among attenders (more than 90 % of whom are of White ethnicity). Although, this figure does reflect the 2001 UK Census, which reported that 89 % and 97 % of English and Welsh residents, respectively, from urban areas were classified as of ‘White’ ethnicity [35].

Despite changes in the most recent Consolidated Standards of Reporting Trials (CONSORT) guidelines, there are very few trials that report the figures required to assess the impact of trial participation on health outcomes and survival [36]. It is difficult to assess who is likely to attend screening if trials do not publish prerecruitment figures and the details of those who are being enrolled remain under-reported [37, 38]. Without adequate reporting of the groups who do and do not participate potential external validity and barriers to screening and treatment cannot be properly assessed. Describing the characteristics of those enrolled and those not enrolled in trials, to ascertain the generalisability of the trial results and to assess the validity of the recruited sample, has been recommended [39].

## Conclusions

We have reported key characteristics that describe those who did and those who did not respond to a single invitation to undergo a PSA test in the ProtecT and CAP RCTs. Overall, there were only minor differences in area-deprivation measures and urban-rural location between those who responded and did not respond to the invitation. Therefore, this is unlikely to affect the generalisability of the ProtecT and CAP trials. Despite restricted access to routine data limiting the comparisons that could be made between responders and nonresponders, this analysis suggests that those who are likely to engage with screening do not differ from those who would not in a meaningful way. Moreover, the characteristics of those who responded are comparable to those observed in other screening trials and add to knowledge about who might accept an invitation to PCa screening, if a programme were to be initiated in England and Wales.

## Abbreviations

CAG: Confidentiality Advisory Group
CAP: Cluster randomised triAl of PSA testing for Prostate cancer
CI: Confidence interval
GP: General practitioner
HVE: Healthy Volunteer Effect
IMD: Index Multiple Deprivation
IQR: Interquartile range
MREC: Multicentre Research Ethics Committee
OR: Odds ratio
PCa: Prostate cancer
PIAG: Patient Information Advisory Group
ProtecT: Prostate testing for cancer and treatment
PSA: Prostate-specific antigen
RCTs: Randomised controlled trials
RUAC: Rural and Urban Area Classification
SD: Standard deviation
UKNSC: UK National Screening Committee
